# Polyunsaturated Fatty Acid Intake and Risk of Lung Cancer: A Meta-Analysis of Prospective Studies

**DOI:** 10.1371/journal.pone.0099637

**Published:** 2014-06-12

**Authors:** Yu-Fei Zhang, Jian Lu, Fei-Fei Yu, Hong-Fang Gao, Yu-Hao Zhou

**Affiliations:** 1 Department of Oncology, Shanghai Seventh People's Hospital, Shanghai, China; 2 Department of Health Statistics, Second Military Medical University, Shanghai, China; 3 Department of Rehabilitation Institute, Shanghai Seventh People's Hospital, Shanghai, China; Geisel School of Medicine at Dartmouth College, United States of America

## Abstract

**Background:**

Studies have reported inconsistent results for the existence of an association between polyunsaturated fatty acid (PUFA) intake and risk of lung cancer. The purpose of this study is to summarize the evidence regarding this relationship using a dose response meta-analytic approach.

**Methodology and Principal Findings:**

We searched the PubMed, EmBase, and Cochrane Library electronic databases for related articles published through July 2013. Only prospective studies that reported effect estimates with 95% confidence intervals (CIs) of lung cancer incidence for greater than 2 categories of PUFA intake were included. We did random-effects meta-analyses of study-specific incremental estimates to determine the risk of lung cancer associated with a 5 g per day increase in PUFA intake. Overall, we included 8 prospective cohort studies reporting data on 1,268,442 individuals. High PUFA intake had little or no effect on lung cancer risk (risk ratio [RR], 0.91; 95% CI, 0.78–1.06; P = 0.230). Furthermore, the dose-response meta-analysis also suggested that a 5 g per day increase in PUFA has no significant effect on the risk of lung cancer (RR, 0.98; 95%CI: 0.96–1.01; P = 0.142). Finally, the findings of dose response curve suggested that PUFA intake of up to 15 g/d seemed to increase the risk of lung cancer. Furthermore, PUFA intake greater than 15 g/d was associated with a small beneficial effect and borderline statistical significance. Subgroup analyses for 5 g per day increment in PUFA indicated that the protective effect of PUFA was more evident in women (RR, 0.94; 95% CI, 0.87–1.01; P = 0.095) than in men (RR, 1.00; 95% CI, 0.98–1.02; P = 0.784).

**Conclusion/Significance:**

Our study indicated that PUFA intake had little or no effect on lung cancer risk. PUFA intake might play an important role in lung cancer prevention in women.

## Introduction

Lung cancer is the leading cause of cancer-related death worldwide for both men and women, and around 1.5 million new cases are diagnosed each year [1]–[2]. For the past few decades, studies have shown that eicosanoids derived from polyunsaturated fatty acid (PUFA) consumption influence many physiological processes, including calcium transport across cell membranes, angiogenesis, apoptosis, cell proliferation, and immune cell function [3]–[5]. Epidemiologic studies have suggested that a healthy diet and lifestyle are critical for prevention of lung cancer [6]–[8]. Dietary fat has been closely related to lung cancer risk. Among subtypes of dietary fat, PUFA is the most promising for inhibiting carcinogenesis and reducing lung cancer risk. However, data on the effect of PUFA intake on subsequent lung cancer morbidity are limited and inconclusive.

The results of a previous prospective study indicated that high PUFA intake was associated with lower lung cancer risk [9]. In contrast, another important study showed that high PUFA intake was associated with greater risk of lung cancer morbidity [10]. Clarifying the optimal daily intake of PUFA is particularly important in the general population, as it has not been definitively determined. Here, we attempted a large-scale examination of the available prospective studies to determine the association between PUFA intake and lung cancer morbidity. We also performed a dose response meta-analysis to quantify the risk of lung cancer with an incremental increase in PUFA intake for the general population.

## Methods

### Data Sources, Search Strategy, and Selection Criteria

This review was conducted and reported according to the Preferred Reporting Items for Systematic Reviews and Meta-Analysis Statement issued in 2009 (Checklist S1) [11]. Any prospective study that examined the relationship between PUFA intake and lung cancer morbidity was eligible for inclusion in our study, and no restrictions were placed on language or publication status (published, in press, or in progress). We searched the PubMed, EmBase, and Cochrane Library electronic databases for articles published through July 2013 using the following search terms: (“fat” OR “fatty acid” OR “docosahexaenoic acid” OR “eicosapentaenoic acid” OR “docosapentaenoic acid” OR “alpha-linolenic acid” OR “polyunsaturated fatty acid” OR “omega-3 fatty acid” OR “n-3 fatty acid” OR “fish” OR “fish oil” OR “seafood” OR “PUFA”) AND (“lung cancer” OR “lung neoplasm” OR “lung carcinoma”) AND (“cohort” OR “cohort studies” OR “nest case-control studies”) AND (“human”). We also conducted manual searches of reference lists from all relevant original and review articles to identify additional eligible studies. The medical subject heading, methods, population, study design, exposure, and outcome variables of these articles were used to identify the relevant studies.

The literature search was independently undertaken by 2 authors (YFZ and HFG) using a standardized approach. Any inconsistencies between these 2 authors were settled by the primary author (YHZ) until a consensus was reached. A study was eligible for inclusion if the following criteria were met: (1) the study had a prospective design (prospective cohort or nest prospective case-control study); (2) the study investigated the association between PUFA intake and risk of lung cancer; and (3) the authors reported effect estimates [risk ratio (RR), hazard ratio (HR), or odds ratio (OR)] and 95% confidence intervals (CIs) for comparisons of high and low PUFA intake (with more than 2 categories of PUFA intake). We excluded all case-control studies because various confounding factors could bias the results.

### Data Collection and Quality Assessment

The following data elements were collected: name of the first author or study group, publication year, country, study design, assessment of PUFA exposure, sample size, age at baseline, percentage of male patients, follow-up duration, effect estimate, endpoints reported, comparison categories, and covariates in the fully adjusted model. We also extracted the numbers of cases per person or per person-year, effect of the different exposure categories, and 95% CIs. For studies that reported several multivariable adjusted RRs, we selected the effect estimate that was maximally adjusted for potential confounders.

The Newcastle-Ottawa Scale (NOS) was used to evaluate methodological quality. The NOS is a comprehensive tool that has been partially validated for evaluating quality of observational studies in meta-analyses [12]–[13]. The NOS is based on the following 3 subscales: selection (4 items), comparability (1 item), and outcome (3 items). A “star system” (range 0–9) has been developed for assessment (Table S1). The data extraction and quality assessment were conducted independently by 2 authors (YFZ and HFG). Information was examined and adjudicated independently by an additional author (YHZ), who referred to the original studies.

### Statistical Analysis

We examined the relationship between PUFA intake and risk of lung cancer on the basis of the effect estimate (RR or HR) and its 95% CI published in each study. We first used the random-effects model [14], [15] to calculate summary RRs and 95% CIs for high PUFA intake compared to low PUFA intake. We subsequently transformed category-specific risk estimates into estimates of the risk ratio (RR) associated with every 5 g per day increase in PUFA intake by use of the method of generalized least-squares for trend estimation [16]. These estimates were calculated from the assumption of a linear relation between the natural logarithm of risk ratio and increasing PUFA intake. We converted fish and fish oil into PUFA, defined 100 g of fish as 2.5 g PUFA and 100 g of fish oil as 30 g PUFA. The value assigned to each PUFA category was the mid-point for closed categories, and the median for open categories (assuming a normal distribution for PUFA intake). We conbined the risk ratios for each 5 g per day increase in PUFA intake by use of random-effect meta-analysis [14]. Unless otherwise stated, we used the most adjusted risk estimate from each study as stated above. We finally conducted a dose response random-effects meta-analysis from the correlated natural log of RRs or HRs across the PUFA intake categories [16], [17]. To derive the dose response curve, we modeled PUFA by using restricted cubic splines with 3 knots at fixed percentiles of 10%, 50%, and 90% of the distribution [16]. This method requires the effect measure with its variance estimate for at least 3 known categories of exposure. Heterogeneity between studies was investigated by using the Q statistic, and we considered P values<0.10 as indicative of significant heterogeneity [18], [19]. Subgroup analyses were conducted for lung cancer on the basis of country, sex, assessment of exposure, and duration of follow-up. We also performed a sensitivity analysis by removing each individual study from the meta-analysis. Several methods were used to check for potential publication bias. Visual inspections of funnel plots for lung cancer were conducted. The Egger and Begg tests were also used to statistically assess publication bias for lung cancer [20], [21]. All reported P values were 2-sided, and P values <0.05 were considered statistically significant for all included studies. Statistical analyses were performed using STATA software (version 12.0; Stata Corporation, College Station, TX, USA).

## Results

**Figure 1:**
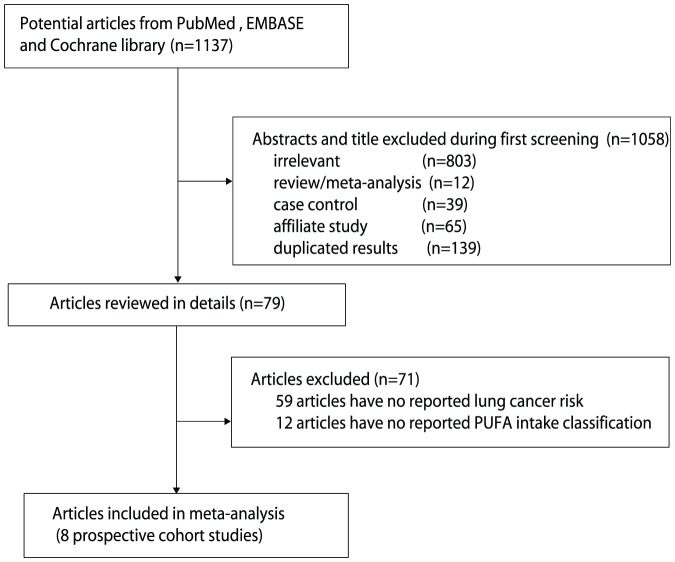
Flow diagram of the literature search and studies selection process.

The results of the study selection process are shown in Figure 1. We identified 1,137 articles in our initial electronic search; 79 remained after exclusion of duplicates and irrelevant studies. After detailed evaluation, 8 prospective studies were selected for the final meta-analysis [9], [10], [22]–[27]. A manual search of the reference lists of these studies did not yield any new eligible studies. General characteristics of the included studies are presented in Table 1.

**Table 1:** Baseline characteristic of studies included in the systematic review and meta-analysis.

Study	Country	Study design	Assessment of exposure	Sample size	Age at baseline	Percentage of male (%)	Effect estimate	Comparison categories	Follow-up (year)	Covariates in fully adjusted model
CR Daniel 2011 [22]	US	Cohort	FFQ	492,186	50–71	59.6	HR	Fish: 21.4 vs 3.6 g/1000 kcal	9.0	Red meat intake, age, sex, education, marital status, family history of cancer, race, BMI, smoking status, PA, MHT in women, and intake of alcohol, fruit, vegetables, and total energy
EV Bandera 1997 [23]	US	Cohort	FFQ	48,000	40–80	57.4	RR	Polyunsaturated fat: tertiles 3 vs tertiles 1	7.0	Age, education, cigarettes/day, years smoking, and total energy intake
P Knekt 1991 [24]	Finnish	Cohort	self-administered questionnaire	4,538	20–69	100	RR	Polyunsaturated fat: tertiles 3 vs tertiles 1	20.0	Age, smoking, and energy intake
K Ozasa 2001 [25]	Japan	Cohort	FFQ	110,792	40–79	38.8	HR	Fish: 1 per/day vs <1–2 per/week	7.0	Age, parents' history of lung cancer and smoking index
MB Veierod [10] 1997	Norway	Cohort	FFQ	51,452	16–56	50.4	RR	Polyunsaturated fat: quartile 4 vs tertiles 1	11.0	Smoking status, gender, age at inclusion, and attained age
I Laake 2012 [26]	Norway	Cohort	FFQ	77,568	35–49	52.0	HR	Fish oil: >2.35 vs <0.85	24.8	Year of birth, energy intake, smoking, BMI, level of physical activity, education level, and sex.
T Takezaki [9] 2003	Japan	Cohort	FFQ	5,885	30 or more	47.5	RR	Fish: 3 or more times per week vs <1 time per week	14.0	Age, sex, occupation, smoking, drinking, exercise habit, and consumption of meat, green-yellow vegetables, and salty/dried fish.
J Linseisen 2011 [27]	Europe	Cohort	Self- administered questionnaire	478,021	25–70	29.8	RR	Fish: >80 g per day vs <9 g per day	8.7	sex, center, and age and adjusted for smoking status, body weight and height, energy intake from fat and energy intake from carbohydrates and protein, intake of alcohol, consumption of fruits and vegetables, PA, and education.

# FFQ: Food Frequency Questionnaire; BMI: body mass index; PA: physical activity.

All 8 included studies were prospective cohort studies (for a total of 1,268,442 individuals). Between 4,538 and 492,186 individuals were included in each study, and follow-up periods ranged from 7.0 to 24.8 years. Two studies were conducted in the United States [22], [23], 4 in Europe [10], [24], [26], [27], and the remaining 2 were conducted in Japan [9], [25]. Study quality was assessed using the NOS (Table S1) [12]. Here we considered a study with a score ≥7 as being of high quality. Overall, 3 studies had a score of 9 [9], [10], [24], 3 studies had a score of 8 [22], [26], [27], and 2 studies had a score of 7 [23], [25].

**Figure 2:**
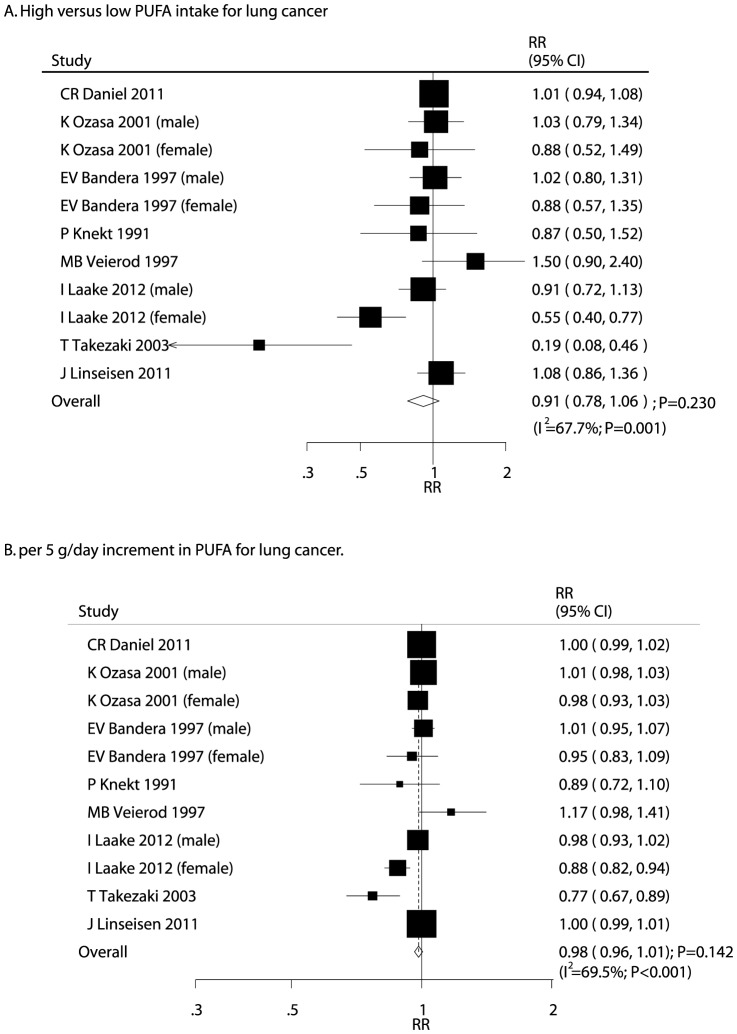
Relative risk estimates of lung cancer for high versus low PUFA intake (A); Dose-response meta-analysis for per 5 g/day increment in PUFA intake for lung cancer (B).

After pooling included studies, the summary RR showed that a high PUFA intake was not associated with lung cancer (RR, 0.91; 95% CI, 0.78–1.06; P = 0.230; Figure 2A), but potential evidence of significant heterogeneity was seen (I^2^ = 67.7%; P = 0.001). The findings of dose-response meta-analysis also suggested that no association with risk of lung cancer per 5 g/day increment of PUFA intake (RR, 0.98; 95%CI: 0.96–1.01; P = 0.142; Figure 2B), heterogeneity between studies was high for lung cancer (I^2^ = 69.5%; P<0.001). As a result, a sensitivity analysis was conducted, and after each study was sequentially excluded from the pooled analysis, the conclusion was not affected by exclusion of any specific study.

**Figure 3:**
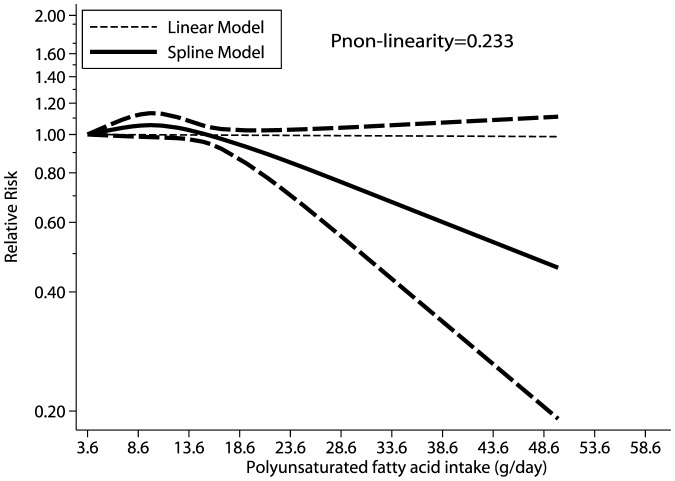
Dose-response relations between PUFA intake and relative risks of lung cancer.

All studies were included in the dose response curve between PUFA intake and incidence of lung cancer. As shown in Figure 3, PUFA intake of 3.6–15.0 g/d seemed to increase the risk of lung cancer. This harmful effect was observed for PUFA intake ≤15 g/d; however, PUFA intake greater than 15 g/d was associated with a small beneficial effect and borderline statistical significance. However, as shown by the P value of nonlinearity (P = 0.233), there was no evidence of a potential nonlinear relationship (Figure 3).

**Figure 4:**
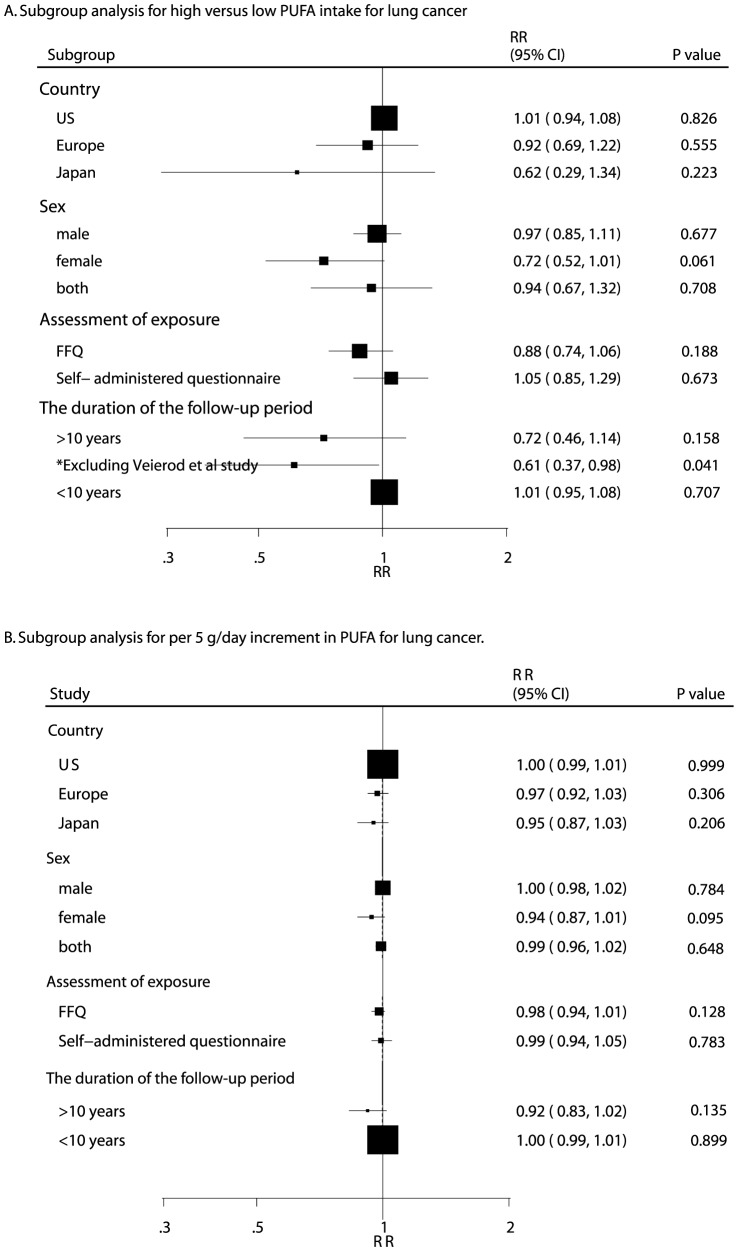
Subgroup analysis for high versus low PUFA intake for lung cancer (A); Subgroup analysis for per 5 g/day increment in PUFA intake for lung cancer (B).

Owing to a P value of <0.10 for heterogeneity testing, we conducted subgroup analyses to minimize heterogeneity among the included studies. Overall, we noted that a high PUFA intake was associated with a reduction in lung cancer risk if the follow-up period was greater than 10 years, when excluding Veierod et al. 's study [10] (RR, 0.61; 95% CI, 0.37–0.98; P = 0.041, Figure 4A). Furthermore, we found that 5 g of PUFA intake increment per day may be a protective factor for lung cancer in women (RR, 0.94; 95% CI, 0.87–1.01; P = 0.095, Figure 4B). No other significant differences in effect were identified between PUFA intake and the risk of lung cancer.

**Figure 5:**
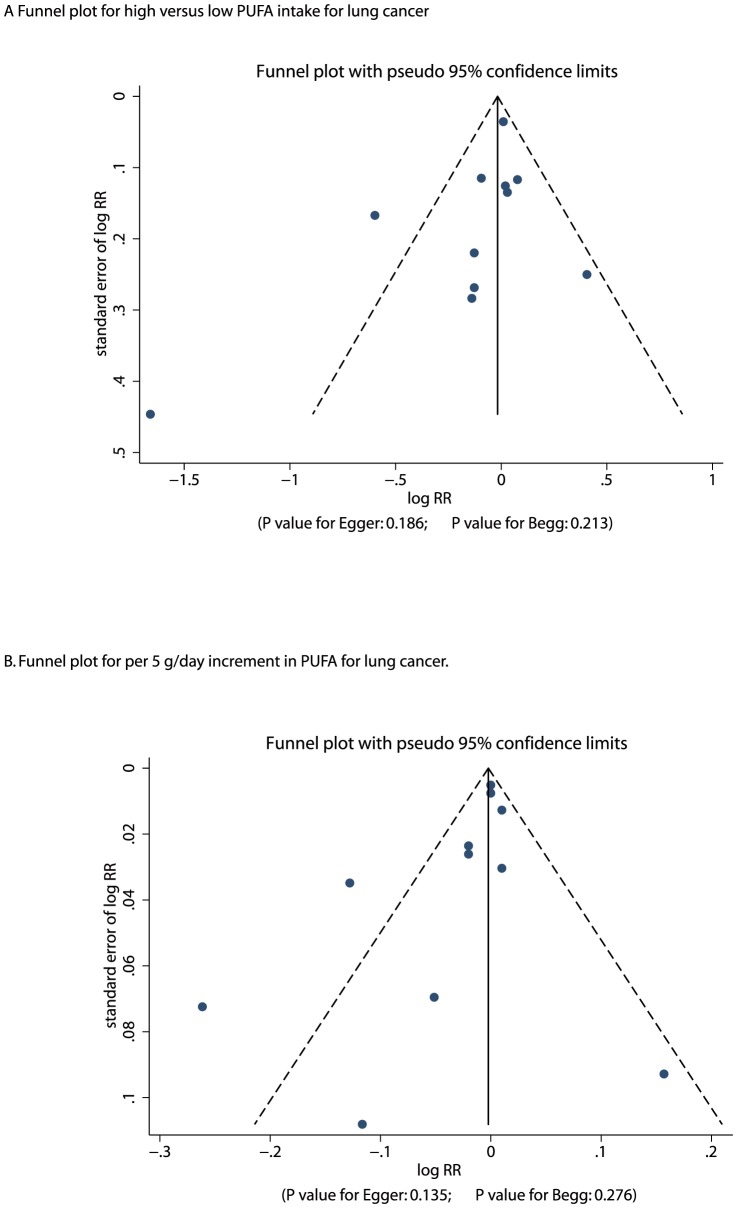
Funnel plot for high versus low PUFA intake for lung cancer (A); Funnel plot for per 5 g/day increment in PUFA intake for lung cancer (B).

A review of funnel plots could not rule out the potential for publication bias for lung cancer (Figure 5). However, the Egger [20] and Begg test [21] results showed no evidence of publication bias for lung cancer (Egger: P = 0.186 for high versus low PUFA intake, and P = 0.135 for per 5 g per day increment in PUFA intake; Begg: P = 0.213 for high versus low PUFA intake, and P = 0.276 for per 5 g per day increment in PUFA intake).

## Discussion

Previous observational studies of the association between PUFA intake and lung cancer risk have been inconclusive. Several case-control studies found a decreased risk with PUFA consumption [28]–[30]. Two other case-control studies failed to find a significant association, although the observed odd ratios were below unity [31], [32]. A positive association was found in 1 study in China, but the subjects consumed fish relatively infrequently and the authors suggested the influence of a potential residual bias [33]. However, various confounding factors in case-control studies could bias the results. Furthermore, the cutoff value for optimal PUFA intake categories differed between studies. Therefore, we conducted a dose response meta-analysis of prospective studies to determine the risk of lung cacner with an incremental increase in PUFA intake.

Our current study was based on prospective studies and explored all possible correlations between PUFA intake and risk of lung cancer. This large quantitative study included 1,268,442 individuals from 8 prospective cohort studies with a broad range of populations. The findings from our current meta-analysis suggest that increased PUFA intake had little or no effect on the incidence of lung cancer. To the best of our knowledge, this is the first time a meta-analysis has evaluated, systematically and quantitatively, the association between intake of PUFA and risk of lung cancer.

Of the 8 studies examined, the majority indicated no association between intake of PUFA and lung cancer incidence, but 2 prospective studies reported a beneficial effect of PUFA in some special populations [9], [26]. In a prospective study of 5,885 individuals, Takezaki et al. found that participants who consumed fish 3 or more times per week had an 81% lower risk of lung cancer than those who consumed fish less than 1 time per week [9]. Laake et al. also suggested that high PUFA intake significantly reduced the risk of lung cancer in women [26]. The pooled results of our meta-analysis were consistent with most studies analyzed –i.e., no evidence of an association between PUFA intake and lung cancer risk was noted. Moreover, we discovered that the pooled RR estimate points were <1 and had a potential trend to deviate to the left. Our dose response curve showed nonsignificant nonlinear relationships between PUFA intake and lung cancer: a low PUFA intake (3.6–15.0 g/d) seemed to increase risk of lung cancer; high PUFA intake (>15 g/d) seemed to have a small beneficial effect on risk of lung cancer. Hence, we suggest there might be a potential protective effect of high PUFA intake on lung cancer incidence; however, this protective effect may not be clinically significant and should be validated by further research.

In our current study, there was no significant difference between increased PUFA intake and the risk of lung cancer. The degree of association may be too low to detect an expected protective effect. Two possible explanations are: (1) different cooking method may moderate the effect of PUFA intake, and (2) different histological or cell types of lung cancer might provide a biased view of the study question. Previous studies showed that consumption of salty or dried fish increased risk of lung cancer [32], [34]. In addition, fish oil rapidly degrades owing to oxidation and other chemical changes when exposed to air, light, heat, and processing, thereby decreasing the concentration of PUFA [35]. In regards to the type of lung cancer, previous studies suggested that high PUFA intake was associated with reduced the risk of adenocarcinomas but not squamous cell or small cell carcinomas [36].

Subgroup analyses indicated that the protective effect of increased PUFA intake was more evident in women than in men. One possible explanation for this could be higher smoking rates among men than among women (i.e., the protective effect of PUFAs on lung cancer risk may be negated by smoking among men). The proportion of current smokers was highest in the lowest category of PUFA intake [26]; it is possible that adjustment for smoking was insufficient in these studies. In never-smokers, Laake et al. found a significant positive association between PUFA intake and lung cancer risk in men, and a positive nonsignificant association in women [26]. Therefore, PUFA intake in men might be too low to detect a protective effect. In addition, we observed that the protective effect of PUFA was more evident in studies with follow-up periods greater than 10 years as compared to those with shorter follow-up periods. The reason for this difference could be that studies with shorter follow-up periods (less than 10 years) did not reach statistical significance owing to low incidence of lung cancer. In addition, only 3 prospective studies had follow-up periods of more than 10 years [9], [24], [26]. This conclusion may be unreliable since smaller cohorts were included in such subset. Therefore, we generated a relative result by comparing high PUFA intake with low PUFA intake, and provided a comprehensive review of the model.

Four strengths of our study should be highlighted. First, only prospective studies were included, which should eliminate selection and recall bias. Second, the large sample size allowed us to quantitatively assess the association of PUFA intake with risk of lung cancer, thus making it more powerful than any individual study. Third, the dose response analysis included a wide range of PUFA intake, which allowed for accurate assessment of the dose relationship between PUFA intake and lung cancer risk. Fourth, no meta-analysis had been performed to date to provide a comprehensive view of the association between PUFA and lung cancer risk.

The limitations of our study are as follows: (1) publication bias is very possible in meta-analyses of published studies; (2) data on histological or cell types of lung cancer and non-smoking subjects were not available so we could not differentiate effects of PUFA intake by histological or cell types of lung cancer; and (3) the analysis used pooled data (individual data were not available), which restricted us from performing a more detailed relevant analysis and obtaining more comprehensive results.

The findings of this study suggest that increased PUFA intake had no significant effect on lung cancer risk. Subgroup analyses suggested that increased PUFA intake might play an important role in lung cancer prevention in women. According to dose response curve, a PUFA intake of more than 15 g/d seemed to lower the risk of lung cancer, but the difference in risk was not statistically significant. Future studies should: (1) focus on specific populations to evaluate strategies for primary prevention of lung cancer; (2) ascertain the specific histological or cell types of lung cancer and analyze effects by type; and (3) take cooking method into consideration when assessing PUFA intake and its effects on clinical outcomes.

## Supporting Information

Table S1 **Quality scores of prospective cohort studies using Newcastle-Ottawa Scale.**
(DOC)

Checklist S1 **PRISMA Checklist.**
(DOC)
