# Supplementary material for: Polyunsaturated Fatty Acid Intake and Risk of Lung Cancer: A Meta-Analysis of Prospective Studies
Source: PLoS One. 2014 Jun 12;9(6):e99637. doi: 10.1371/journal.pone.0099637 (PMC4055702; doi:10.1371/journal.pone.0099637)
Supplement: Table S1 — Quality scores of prospective cohort studies using Newcastle-Ottawa Scale. (DOC) [file pone.0099637.s001.doc]

Table S1. Quality scores of prospective cohort studies using Newcastle-Ottawa Scale.

| Study | Selection | | | | Comparability | Outcome | | | NOS |
| --- | --- | --- | --- | --- | --- | --- | --- | --- | --- |
|  | Representativeness of the exposed cohort | Selection of the non exposed cohort | Ascertainment  of PUFA intake | Demonstration that outcomes was not present at start of study | Comparability on the basis of the design or analysis | Assessment of outcome | Adequate follow-up duration | Adequate follow-up rate | Overall score |
| CR Daniel 2011 [22] | 1 | 1 | 1 | 1 | 2 | 1 | 1 | 0 | 8 |
| EV Bandera 1997 [23] | 1 | 1 | 1 | 1 | 1 | 1 | 1 | 0 | 7 |
| P Knekt 1991 [24] | 1 | 1 | 1 | 1 | 2 | 1 | 1 | 1 | 9 |
| K Ozasa 2001 [25] | 1 | 1 | 1 | 1 | 1 | 1 | 1 | 0 | 7 |
| MB Veierod [10] 1997 | 1 | 1 | 1 | 1 | 2 | 1 | 1 | 1 | 9 |
| I Laake 2012 [26] | 1 | 1 | 1 | 1 | 1 | 1 | 1 | 1 | 8 |
| T Takezaki [9] 2003 | 1 | 1 | 1 | 1 | 2 | 1 | 1 | 1 | 9 |
| J Linseisen 2011 [27] | 1 | 1 | 1 | 1 | 2 | 1 | 1 | 0 | 8 |
